# A Retrospective Study on the Morphology of Posterior Malleolar Fractures Based on a CT Scan: Whether We Ignore the Importance of Fracture Height

**DOI:** 10.1155/2020/2903537

**Published:** 2020-07-29

**Authors:** Zhifeng Wang, Chengjie Yuan, Genrui Zhu, Xiang Geng, Chao Zhang, Jiazhang Huang, Xin Ma, Xu Wang

**Affiliations:** Department of Orthopedics, Huashan Hospital, Fudan University, No. 12, Middle Wulumuqi Road, Jingan District, Shanghai, China

## Abstract

**Objective:**

The aim of this study was to investigate the respective correlation between the height (*H*) of a posterior malleolar fracture (PMF) and the involved area (*S*) of an articular surface and the presence of “die-punch.”

**Methods:**

Patients with closed posterior malleolar fractures admitted to our hospital from January 2015 to December 2017 were selected, with complete X-ray and 3D reconstruction CT imaging data. The gender, age, injured side, and surgical fixation methods of the patients were recorded. A preoperative ankle CT scan was performed, and the images were viewed through the PACS (Picture Archiving and Communication Systems). Simultaneously, the involved joint surface area (*S*) by the posterior malleolar fracture was measured, as well as the proportion of the fracture area to the total ankle joint area. On the sagittal reconstruction CT images, the height (*H*) of the posterior malleolar fracture was measured to compare the correlation between the height of the fracture and the area of the fracture, as well as the area ratio. Besides, according to the presence or absence of “die-punch,” patients were divided into two groups: A and B. And each group was further divided into three subgroups according to age (16-39 years old, 40-59 years old, and ≥60 years old). The statistical differences in the height of fracture between the subgroups were compared.

**Results:**

A total of 48 patients, aged 16-82 years, with an average age of 48.9 years, were included in this study, including 13 males and 35 females. There were 20 cases of left ankle injury and 28 cases of right ankle injury. The average height of the posterior malleolar fractures was 18.19 mm, the average area of the fracture was 202.28 mm^2^, and the average ratio of the fracture area to the total articular surface area was 17.84%. Besides, die-punch was seen in 27 cases and not in 21 cases. The average height of fractures was 21.33 ± 5.38 mm in group A1, 14.38 ± 9.01 mm in group B1, 18.30 ± 7.95 mm in group A2, 14.48 ± 5.37 mm in group B2, 26.26 ± 6.73 mm in group A3, and 12.77 ± 3.07 mm in group B3.

**Conclusion:**

The height (*H*) of the posterior malleolar fractures is positively correlated with the fracture area (*S*) and the fracture area ratio (FAR). The posterior malleolar fractures with “die-punch” tend to have a greater average height than that without “die-punch.” In clinical work, orthopedic surgeons should not only pay attention to the size of the posterior malleolus fracture but also value its height, which hopefully could provide insight into the treatment and prognosis of PMF patients.

## 1. Introduction

An ankle fracture is common in clinical work, accounting for about 3.9% of all fractures. It has been reported in the previous literature that posterior malleolus fractures account for 7% to 44% of all ankle fractures [1–3]. Simple posterior malleolus fractures are rare, accounting for about 0.5%-1% of all fractures [4, 5]. The posterior malleolus where the posterior tibiofibular ligament is attached is an important structure to maintain the stability of the ankle.

Ankle fractures that did not involve the posterior malleolus had a 30 percent lower rate of traumatic arthritis than posterior malleolus fractures [6]. The ankle fracture involving the posterior malleolus often has poor prognosis and functional recovery. Clinical studies have shown that the presence of a PMF is important as a prognostic factor or functional outcome in the treatment of ankle fractures [2, 7–9].

The classification systems of the posterior malleolus fracture are the following types: AO classification, Heim classification, Haraguchi classification, and Bartoníček classification. The first two types are based on X-ray, and the latter two are classified according to CT images. However, most studies had proven that using plain radiographs provides limited understanding of the pathoanatomy of PMF. CT imaging can evaluate the exact shape of the posterior malleolus fracture. Some scholars [10] recommended that all patients with trimalleolar fractures should be fully evaluated by ankle CT before operation. The first classification of the posterior malleolus fracture based on CT scanning was put forward by Haraguchi et al. [11] in 2006. However, this classification only observed and analyzed the cross-sectional images and did not perform 2D or 3D CT reconstruction. In 2015, Bartoníček et al. [12] analyzed 141 consecutive patients suffering from a posterior malleolus fracture with 3D reconstruction of CT images and proposed a new classification.

The posterior malleolar fragment of an ankle fracture can have various shapes depending on the injury mechanism. Also, the posterior malleolus fracture is often accompanied by posterior dislocation of an ankle joint, cartilage lesions, and even die-punch. Nowadays, a die-punch fracture is known as a special intra-articular fracture, mechanically a depression fracture of the distal tibia caused by a vertical load. The posterior malleolus fracture often involves the weight-bearing articular surface of the tibiotalar joint, resulting in the impact and compression. At this time, some small bone fragments are often embedded between the fracture sutures; this sign is called “die-punch.” For the poor prognosis and functional recovery, more and more orthopedic doctors pay attention to the PMF. Current imaging studies of the posterior malleolus mainly focus on the size of the fracture and the affected area of the articular surface. Yi et al. [13] suggested that a better understanding of morphological characteristics of the PMF depending on the mechanism of the injury could be useful to surgeons on planning the fixation of the PMF and may provide a basis for prognosis. Previous studies focused on the affected area of the ankle joint surface but paid less attention to the height of the fracture fragment and the correlation between the height of PMF and the appearance of “die-punch.”

The purpose of this study was to evaluate the correlation between the height of PMF and the affected articular surface area or the area ratio on the basis of comprehensive CT. In addition, we aimed to investigate the relationship between the height of PMF and the appearance of “die-punch” and provide insight for orthopedic surgeons into addressing PMFs, especially those with “die-punch.”

## 2. Methods

We retrospectively reviewed consecutive patients who underwent surgery for a posterior malleolus fracture from January 2015 to December 2017. The following are the exclusion criteria in this study: patients who were below 16 years old, had congenital deformity, had fractures without a PMF, and had a pathological fracture. We retrospectively analyzed 3D reconstruction CT data of 48 patients (48 ankles) who underwent surgery for an ankle fracture including PMF.

### 2.1. Experimental Equipment

The CT examination equipment used is Philips Brilliance 256-row spiral CT (Philips Company, Netherlands).

### 2.2. Measurement

**Figure 1:**
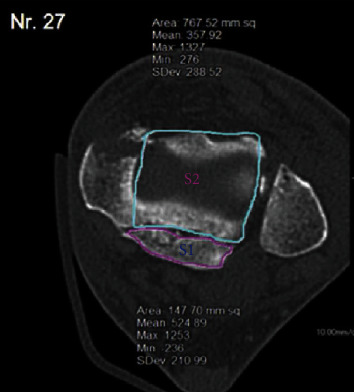
Measurements of the fracture area. The area of the fracture block (S1), which is the purple area, was measured with Extended Brilliance Workspace software. Meanwhile, the area of the residual articular surface (S2), which is the turquoise area, was measured. Then, the fracture area ratio (FAR = S1/(S1 + S2)) was calculated.

**Figure 2:**
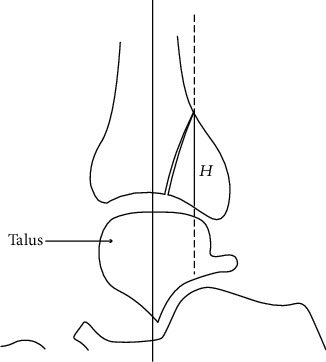
Measurement of the fracture height (*H*). The largest distance from the apex of the fragment to the point which is crossed by the dotted line and the articular surface on consecutive sagittal reconstruction views is defined as the fragment height (*H*).

The ankle CT was observed and measured by the image archiving and communication system (PACS) of Huashan Hospital affiliated to Fudan University. The main contents of observation or measurement were as follows: the area of the fracture block (S1) and the area of the residual tibial-talus articular surface (S2) were measured with Extended Brilliance Workspace software, and the proportion of the fracture area to the total area of the distal tibial articular surface (FAR = S1/(S1 + S2)) was calculated, as shown in Figure 1; the height of the fracture block, that is, the intersection of the straight line parallel to the tibial axis and the articular surface, was measured, and the distance between the two points was recorded as *H* (mm), as shown in Figure 2, whether there was “die-punch” in ankle cross-sectional CT images.

We measured the height and area of fracture and then calculated the fracture area ratio (FAR) in all patients to compare the fracture height (*H*) with the fracture area (S1) and fracture area ratio (FAR). Moreover, all the patients were divided into two groups: group A and group B according to whether there is “die-punch” on CT images. Each group was further divided into three subgroups depending on their ages (16-39 years old, 40-59 years old, and ≥60 years old). The height of fracture blocks in each group and each subgroup was statistically analyzed.

## 3. Statistical Analysis

The linear regression analyses were used to compare the fracture height (*H*), fracture area (S1), and fracture area ratio (FAR) measurements in all patients. The double-factor ANOVA was used to compare the height difference in PMF morphology of the ankle fractures between group A and group B. A *P* value of <0.05 was considered statistically significant, and the statistical analysis was conducted using GraphPad Prism 7.0 (GraphPad Software, La Jolla City, California, USA).

This study was completed with appropriate institutional review board approval.

## 4. Results

A total of 48 patients, aged 16-82 years, with an average age of 48.9 years, were included in this study, including 13 males and 35 females. There were 20 cases of left ankle injury and 28 cases of right ankle injury. According to the Haraguchi classification of the posterior malleolus fracture, there were 25 cases of type I, 18 cases of type II, and 5 cases of type III. The average height of the fracture was 18.19 mm, and the average area was 202.28 mm^2^. The average fracture area ratio was 17.84%. “Die-punch” was visible in 27 patients and not in 21 patients. The average height of fracture was 21.33 ± 5.38 mm in group A1, 14.38 ± 9.01 mm in group B1, 18.30 ± 7.95 mm in group A2, 14.48 ± 5.37 mm in group B2, 26.26 ± 6.73 mm in group A3, and 12.77 ± 3.07 mm in group B3.

**Figure 3:**
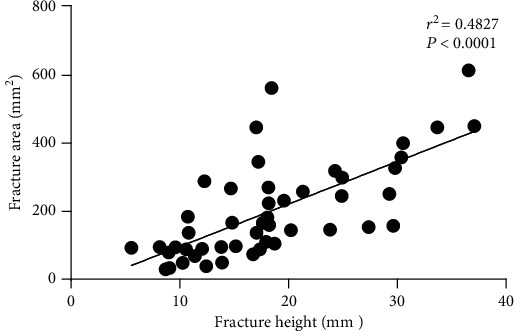
There is a significant positive correlation between the fracture height (*H*) and the fracture area (S1).

**Figure 4:**
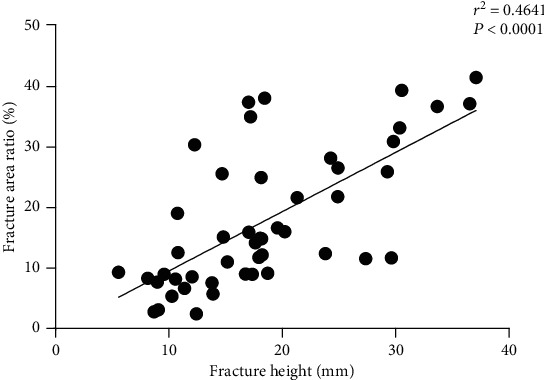
There is a significant positive correlation between the fracture height (*H*) and the fracture area ratio (FAR).

The height of fracture (*H*) was positively correlated with the area of fracture (S1), with a correlation coefficient of 0.4827 (*P* < 0.0001), as well as the fracture area ratio (FAR), whose correlation coefficient was 0.4641 (*P* < 0.0001). The results are shown in Figures 3 and 4.

There was a statistically significant difference in the height (*H*) of fracture blocks between group A1 and group B1 (*P* = 0.0006). There was a significant difference in the height of fracture blocks between the A2 group and the B2 group (*P* = 0.0478 < 0.05). There was also a statistically significant difference in the fracture block height (*H*) between the A3 group and B3 group (*P* < 0.0001).

**Figure 5:**
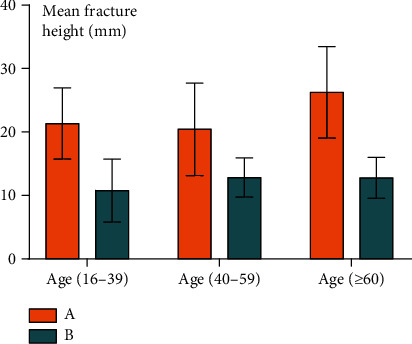
The mean height of fracture in each subgroup. A: group with “die-punch”; B: group without “die-punch.”

**Figure 6:**
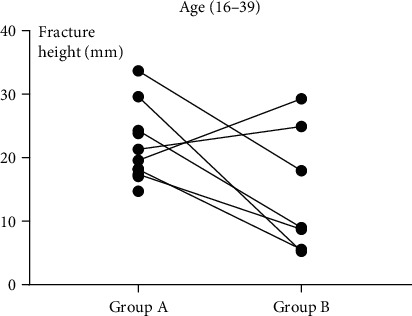
In subgroup 1 (16-39 years old), the height of the fracture with “die-punch” was significantly higher than that without “die-punch.” A: with “die-punch”; B: without “die-punch.”

**Figure 7:**
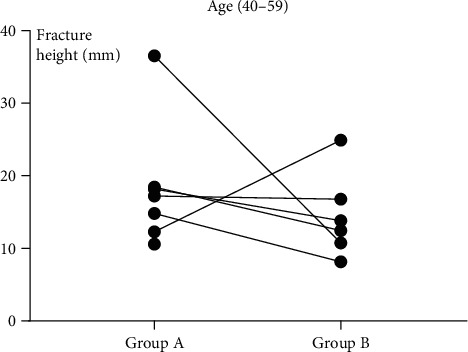
In subgroup 2 (40-59 years old), the height of the fracture with “die-punch” was significantly higher than that without “die-punch.” A: with “die-punch”; B: without “die-punch.”

**Figure 8:**
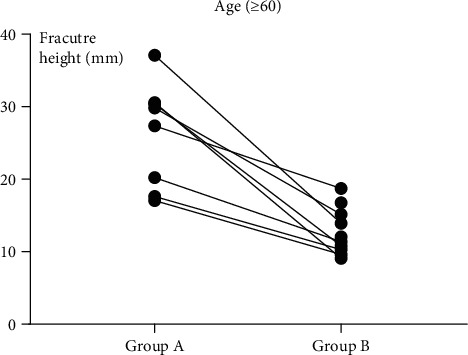
In subgroup 3 (≥60 years old), the height of the fracture with “die-punch” was significantly higher than that without “die-punch.” A: with “die-punch”; B: without “die-punch.”

The fracture height (*H*) measurements in the six subgroups are shown in Table 1. The mean fracture height (*H*) was significantly larger in group A than in group B regardless of subgroup (*P* = 0.024, *P* = 0.017, and *P* = 0.006). These results are shown in Figures 5–8.

## 5. Discussion

The posterior malleolus (PM) was described as the “posterior lip of the distal tibia,” “posterior rim of the distal tibia,” or “posterior margin” in the previous literatures. The posterior malleolus fracture (PMF) is a lesion involving the posterior tibial plafond, including extra-articular osseous avulsions, posterolateral triangular fragment, and impaction of the entire posterior lip [10]. The medial part of PM is separated from the medial malleolus by the malleolar groove containing the posterior tibial tendon. The lateral half of PM is formed by a marked bony prominence, the posterior tubercle of the distal tibia that also forms the posterior part of the fibular notch (incisura fibularis tibiae). In addition, the posterior malleolus is attached by the inferior posterior tibiofibular ligament. The inferior posterior tibiofibular ligament runs laterally and obliquely, ending at the posterior prominence of the distal fibula. It has been reported in the literature that the inferior posterior tibiofibular ligament can provide 42% of the stability of the inferior tibiofibular joint [14].

The fracture that only occurred in the posterior malleolus is rare. Currently, quite a few scholars [15–17] believe that the injury mechanism of the posterior malleolus fracture is the rotation violence applied to the distal tibia, which is closely related to the position of the ankle and the direction of violence. Indirect causes of PMF include soft tissue factors. When the ankle is suddenly subjected to rotation violence, it acts on the posterior malleolus through the inferior posterior tibiofibular ligament, the inferior tibiofibular transverse ligament, joint capsule, etc., and the tear of soft tissue structure is likely to lead to avulsion fracture. Therefore, posterior malleolus fractures are often accompanied by lateral malleolus fractures or/and medial malleolus fractures. All the 48 patients with posterior malleolus fractures included in this study were not merely fractured in the posterior malleolus.

Although there have been many clinical and biomechanical studies about posterior malleolus fractures in recent years, the surgical indications remain controversial. Currently, the consensuses of indication for fixation of PMF are as follows: fragments involving 25–33% of the articular surface, fracture displacement greater than 2 mm, accompanied by ankle joint instability, and posterior dislocation of the talus [18–20]. In 1960, McLaughlin [21] found that when the posterior malleolus fracture involved more than 25% of the articular surface, all patients developed the subluxation of the talus to some extent after active conservative treatments, eventually leading to traumatic arthritis and some other complications. For the first time, he proposed that the fragments involving more than 25% of the articular surface should be fixed, which became one of the most widely accepted indications of PMF. In a previous study [22], the area of articular involvement for the tibial plafond was significantly associated with the contact area of the tibiotalar joint. When the posterior malleolus fracture involved more than 25% of the articular surface, the contact area decreased by 4%~35%. The dominant view in the clinical treatment of posterior malleolus fractures is that open reduction and internal fixation are recommended when the posterior malleolus fracture involves more than 25% to 33% of the articular surface. However, there is a lack of high-level evidence to substantiate its reliability [23]. Some authors hold the opposite views. Through the investigation in a series of 38 patients with PMF involving larger than 25% of the articular surface, among whom 15 were treated with open reduction internal fixation and 23 with closed reduction, Harper et al. [24] suggested that if the anatomic reduction through closed reduction of PMF could be achieved, there were no statistical differences in clinical outcomes between the two groups. Moreover, De Vries et al. [19] investigated 13 patients with PMF involving larger than 25% of the articular surface, with an average follow-up of 13 years, and showed that there was no significant difference in prognosis between the surgical and nonsurgical groups and one patient had a satisfied outcome even though the fracture involved larger than 49% of the articular surface. The results of our present study showed that the average PMF area was 202.28 mm^2^, accounting for 17.84% of the t5otal articular surface. All the 48 patients were treated surgically, and 33 patients underwent ORIF of the posterior malleolus, among which 5 patients were fixed with plates and 28 patients were fixed with screws. In addition, some authors [7, 15, 25] believed that the smaller posterior malleolus fracture should not be ignored, because it might lead to ankle instability or even degeneration. Moreover, the fixation of the posterior malleolus fracture is also conducive to maintaining the stability of syndesmosis [19, 23, 26]. Langenhuijsen et al. [7] believed that the anatomical reduction and internal fixation were required if the posterior ankle fracture involved more than 10% of the articular surface and transferred to >1 mm, so as to restore the integrity of the articular surface and avoid ankle arthritis. The relationship between the area of posterior malleolus fractures and its surgical indications still remains controversial.

The emergence of “die-punch” often means that it is accompanied by vertical axial violence. When the fracture is caused by the combination of rotational violence with axial violence, it is difficult to visualize a simple fracture on imaging due to the stress in multiple directions, and some small bone fragments are often embedded between the fracture sutures, which will hinder the reduction and further affect the prognosis. Some authors [27] referred to this type of fracture as a posterior pilon fracture, which, unlike a pilon fracture, was caused by low-energy vertical axial violence. Amorosa et al. [28] believed that the posterior pilon fracture was mainly caused by the vertical axial violence. The posterior pilon fracture was caused by relatively low-energy violence, and most of its fracture sutures were coronal [29].

Several surgical approaches are available for the treatment of PMFs. In cases in which small osteochondral fragments may interfere with anatomic reduction or become loose bodies or articular impaction is recognized, then it is advisable to approach the fracture site and address this before attempting reduction and fixation of PMFs. The posteromedial or posterolateral surgical approaches readily enable surgeons to address these components of the injury. The posteromedial approach is appropriate for a posteromedial fragment and allows concomitant treatment of the medial malleolus [28, 30, 31]. The posterolateral approach has gained much popularity and allows good visualization of the posterolateral malleolar fragment [28, 32–35]. Furthermore, concomitant treatment of the fibula fracture is easily performed. Surgeons should focus on restoring ankle joint structural integrity, that is, restoring articular congruity, correcting posterior talar translation, addressing articular impaction, removing osteochondral debris, and achieving syndesmotic stability. Surgeons should familiarize themselves with posterolateral and posteromedial approaches [36].

In this study, our patients are divided into two groups according to the presence or absence of “die-punch” and further divided into three subgroups according to age (16-39, 40-59, and ≥60 years old, respectively). We compared the height of posterior malleolus fractures between the corresponding subgroups. The results of our present study showed that the average heights of posterior malleolus fractures with “die-punch” were significantly greater than those without “die-punch” in all corresponding subgroups. The results indicated that it tended to be due to more vertical axial violence occurring with the presence of “die-punch.” Given the mechanism of the ankle injuries, the greater the vertical axial violence to the posterior malleolus, the more parallel the fracture sutures are to the axis of the tibia, resulting in a higher posterior malleolus fracture. Our results were consistent with the injury mechanism, and the double-factor ANOVA was conducted to investigate the correlation between the height of the posterior malleolus fracture and the occurrence of “die-punch,” laying a foundation for the subsequent biomechanical studies.

The posterior malleolus fracture has become one of the hot topics in the field of foot and ankle surgery in recent years, and it is especially important to grasp the surgical indications. In the last few decades, surgical treatment for the posterior malleolus fracture was usually based on the size of the fracture in CT images. Most surgeons believed that open reduction and internal fixation are necessary when the posterior malleolus fracture involved more than 25% to 33% of the articular surface. However, the area of the posterior malleolus fracture is one of the imaging parameters, and orthopedic surgeons often overlook the importance of the height of the fracture. A die-punch fracture usually occurred on patients who encountered very high energy axial loading forces, which resulted in articular comminution or collapse [37]. Our results showed a significant correlation between the height of PMF and the presence of die-punch. That is, the fracture height, as well as its area, is an important prognostic factor, and it can also reveal the characters of the violence and determine the type of ankle injuries. Furthermore, the fracture height may help guide surgeons with regard to fixation requirements for PMFs [38]. To some degree, the height of fracture can provide effective diagnostic information; meanwhile, it may be helpful to surgical interventions, such as the surgical indications, the incisions, and the internal fixations. It is becoming evident that fragment size should not be the only factor to dictate treatment, and surgeons should also focus on the fracture height.

The limitations of our study should be mentioned. Firstly, this was designed as a retrospective, nonrandomized study, which was associated with selection bias and data inaccuracy. Secondly, our enrolled patients were not further divided into groups with simple PMF or bimalleolar/trimalleolar fractures with PMF. And the differences in the issues of the height and fractured area between simple PMF and bi-/trimalleolar fractures were not clear. Further studies are necessary to clarify these differences. Thirdly, due to the small sample size, there is a possibility of statistical error. Future studies with large sample size and randomized design are required to verify our results.

## 6. Conclusion

The height (*H*) of the posterior malleolar fractures is positively correlated with the fracture area (*S*) and the fracture area ratio (FAR). The posterior malleolar fractures with “die-punch” tend to have a greater average height than those without “die-punch.” In clinical work, orthopedic surgeons should not only pay attention to the size of the posterior malleolus fracture but also value its height, which hopefully could provide insight into the treatment and prognosis of PMF patients.

## Figures and Tables

**Table 1:** Fracture height *H* (mm) in each subgroup.

	A1	B1	A2	B2	A3	B3
1	18.15	5.56	17.22	16.78	30.37	10.81
2	29.63	5.25	18.18	13.82	29.8	15.16
3	17.37	8.71	18.45	12.44	30.54	9.07
4	33.68	17.95	36.55	10.76	20.23	11.39
5	21.33	24.9	12.28	24.93	17.04	9.60
6	19.59	29.28	14.81	8.15	27.36	18.72
7	24.28	8.98	10.58		37.09	13.90
8	18.05				17.63	10.28
9	14.71					16.76
10	23.82					12.05
11	18.23					
12	17.06					
*x* ± *σ*	21.33 ± 5.38	14.38 ± 9.01	18.30 ± 7.95	14.48 ± 5.37	26.26 ± 6.73	12.77 ± 3.07

## Data Availability

The datasets generated during and/or analyzed during the current study are available from the corresponding author on reasonable request.
